# TRAP-Induced Platelet Reactivity Is Inhibited by Omega-3 Fatty Acid-Derived Prostaglandin E3 (PGE3)

**DOI:** 10.3390/biomedicines12122855

**Published:** 2024-12-16

**Authors:** José-Miguel Osete, Faustino García-Candel, Francisco-José Fernández-Gómez, Miguel Blanquer, Noemí M. Atucha, Joaquín García-Estañ, David Iyú

**Affiliations:** 1 Department of Physiology, University of Murcia, 30120 Murcia, Spain; josemiguel.osete@um.es (J.-M.O.); ntma@um.es (N.M.A.); jgestan@um.es (J.G.-E.); 2 Servicio de Hematología, Hospital Clínico Universitario Virgen de la Arrixaca, 30120 Murcia, Spain; fausgarcia@um.es; 3 Instituto Murciano de Investigación Biosanitaria (IMIB)—Arrixaca, Unidad de Trasplante Hematopoyético y Terapia Celular, 30120 Murcia, Spain; miguelblanquer@um.es; 4 Department of Pharmacology, University of Murcia, 30120 Murcia, Spain; franciscojose.fernandez@um.es; 5 Department of Medicine, University of Murcia, 30120 Murcia, Spain

**Keywords:** prostaglandin E3, thrombin, EP3 and EP4 receptors, platelet function, P-selectin, VASP phosphorylation

## Abstract

**Background:** Prostaglandins are naturally occurring local mediators that can participate in the modulation of the cardiovascular system through their interaction with Gs/Gi-coupled receptors in different tissues and cells, including platelets. Thrombin is one of the most important factors that regulates platelet reactivity and coagulation. Clinical trials have consistently shown that omega-3 fatty acid supplementation lowers the risk for cardiovascular mortality and morbidity. Since omega-3 fatty acids are the main precursors of PGE3 in vivo, it would be relevant to investigate the effects of PGE3 on Thrombin Receptor Activating Peptide (TRAP-6)-induced platelet reactivity to determine the receptors and possible mechanisms of action of these compounds. **Methods:** We have measured platelet aggregation, P-selectin expression, and vasodilator-stimulated phosphoprotein (VASP) phosphorylation to evaluate platelet reactivity induced by TRAP-6 to determine the effects of PGE3 on platelet function. **Results:** We assessed the ability of DG-041, a selective prostanoid EP3 receptor antagonist, and of ONO-AE3-208, a selective prostanoid EP4 receptor antagonist, to modify the effects of PGE3. PGE3 inhibited TRAP-6-induced platelet aggregation and activation. This inhibition was enhanced in the presence of a Gi-coupled EP3 receptor antagonist and abolished in the presence of a Gs-coupled EP4 receptor antagonist. The effects of PGE3 were directly related to changes in cAMP, assessed by VASP phosphorylation. **Conclusions:** The general effects of PGE3 on human platelet reactivity are the consequence of a balance between activatory and inhibitory effects at receptors that have contrary effects on adenylate cyclase. These results indicate a potential mechanism by which omega-3 fatty acids underlie cardioprotective effects.

## 1. Introduction

Cardiovascular diseases rank as the primary global causes of mortality, significantly impacting overall well-being. In this regard, ischemic heart disease and ischemic stroke are currently leading the global ranking of cardiovascular deaths by cause [1]. In both situations, the involvement of platelet function plays a pivotal role in the pathophysiology of the process [2].

Prostaglandins are naturally occurring local mediators that act on a considerable diversity of organs and cells, including platelets [2]. Different research findings suggest that prostaglandins of the E-series (PGE1, PGE2, and PGE3) might have clinical significance in relation to the cardiovascular system [3,4]. As an example, there have been reports indicating that the anti-atherosclerotic effects of the polyunsaturated fatty acid dihomo γ-linolenic acid may be mediated by PGE1 [5]. Furthermore, research conducted in mice has demonstrated that PGE2 is released from atherosclerotic plaques, and, by acting through the EP3 receptors, may play a role in platelet thrombus formation [6]. In this context, a selective EP3 antagonist, DG-041, was developed as an antithrombotic agent [7,8]. 

Another prostaglandin of the E-series that has been recognized is PGE3, an omega-3 fatty acid-derived compound, which has been reported to modulate the function of endothelial cells [9,10] and have protective and anticancer effects [11,12], although there is not enough data available about its effects on platelets [2], apart from a study by Iyu et al. [13] that revealed a reduced platelet aggregation (PAF-induced) and expression of plasma membrane P-Selectin (U46619-induced) when platelets of human origin were treated with PGE3.

Nevertheless, several clinical trials have consistently shown that omega-3 fatty acid supplementation provides protection from cardiovascular disease [9] and that icosapent ethyl, a highly purified ethyl ester of Eicosapentaenoic acid (EPA), reproducibly reduced cardiovascular events and the progression of atherosclerosis [14]. This can be seen in different studies, such as REDUCE-IT [15], EVAPORATE [16], and JELIS [17]. Other studies have exposed that omega-3 fatty acids may enhance the antiplatelet effect of prostaglandins and confer cardioprotection against arrhytmias [18]. In addition, they have been shown to inhibit platelet function [19], and it has been recently reported that omega-3 fatty acids generate the pro-resolving mediators maresins, protectins, and resolvins that are able to restore homeostasis during acute and chronic disease, including atherosclerosis [20,21]. Adding to that, resolving E1, an omega-3 fatty acid-derived compound, is capable of regulating collagen-induced platelet aggregation [22]. Since EPA is the main precursor of PGE3 in vivo [2], exploring the impact of PGE3 on platelets could be valuable in understanding the potential mechanisms of action of these compounds.

It has been widely documented that PGE1’s and PGE2’s overall impact on platelet function seems to stem from a delicate interplay between their effects at the receptors that activate (Gi) and inhibit (Gs) platelet action: IP for PGE1 and EP4 for PGE2 [23,24,25,26]. Nevertheless, there is minimal documentation available reporting the receptors that mediate the actions of PGE3 on platelet function. One of the few examples, published by Iyú et al., demonstrated that, like PGE2, PGE3 interacts with EP3 and EP4 receptors, but not with the IP receptor. Therefore, the overall effects of PGE3 on platelet function, like those of PGE1 and PGE2, reflect a balance between effects at both activatory and inhibitory receptors.

The balance and expression between EP3 and EP4 receptors may constitute a pivotal role for the cardiovascular system, since it has been reported that the overexpression of EP3 receptor reduces cardiac function under basal conditions and that this is exacerbated after myocardial infarction [27], whereas the overexpression of EP4 improves cardiac function after a myocardial infarction [28]. These changes in the expression of EP3/EP4 receptors may influence the ability of prostaglandins to modulate platelet function. Also, it is important to highlight that the generation of thrombin in coagulation plays a central role in the functioning of hemostasis [29], especially in the context of a platelet-based coagulation where platelets provide a membrane substrate for the prothrombinase complexes, resulting in massive thrombin generation that places thrombin in a pivotal role to modulate platelet reactivity and coagulation [30].

In this study, we have used a pharmacological approach to gain more information on the role of various prostanoid receptors as modulators of platelet function. All experiments were performed using platelets from healthy human volunteers. We performed measurements of platelet aggregation and P-selectin expression to assess platelet function induced by thrombin receptor activating peptide (TRAP-6). We also assessed the ability of selective receptor antagonists to modify the effects of PGE3 on platelet function. The antagonists were the EP3 antagonist DG-041 [7,8] and the EP4 antagonist ONO-AE3-208 [23,24]. As well as studies of platelet function, we also determined the effects of PGE3 and the various antagonists on the levels of phosphorylated vasodilator-stimulated phosphoprotein (VASP), which can be used as a surrogate marker of changes in cAMP levels in platelets [25,26,31,32]. In this regard, it has been established that VASP phosphorylation measurements correlate with changes in cAMP and provide a reliable way of monitoring changes in adenylate cyclase activity in cardiovascular cells [32,33].

The results demonstrate that the overall effects of PGE3 on platelet function reflect the balance between effects at EP3 versus EP4 receptors. The occupation of EP4 receptors leads to the inhibition of platelet function, brought about by thrombin via increasing the level of cAMP, and indicates that the inhibitory effects of PGE3 are mediated by this receptor. In contrast, the interaction with EP3 receptor may potentiate platelet function by lowering cAMP. In general, the ability of PGE3 to inhibit thrombin-induced platelet reactivity may be one the mechanisms by which the cardiovascular benefits of dietary omega-3 fatty acids are conferred.

## 2. Materials and Methods

### 2.1. Materials

The EP4 receptor antagonist ONO-AE3-208 and the EP3 receptor antagonist DG-041 were obtained from Biotechne^®^ Tocris (Bristol, UK). Thrombin Receptor Activating Peptide 6 (TRAP-6) was obtained from Sigma-Aldrich (San Luis, MO, USA). PGE3 was purchased from Cayman Chemical (Ann Arbor, MI, USA). PGE3 was prepared in ethanol and diluted in saline. DG-041 and ONO-AE3-208 were dissolved in dimethyl sulfoxide (DMSO) and diluted in saline. DG-041 was always protected from light. Appropriate vehicle controls were always used in the experimental studies that were performed. CD61-PerCP was purchased from Becton Dickinson (Franklin Lakes, NJ, USA) and CD62P-FITC from Serotec (Bio-Rad: Formerly AbD Serotec. Hercules, CA, USA). For the aggregation and P-Selectin expression measurements, AGGFixA/AGGFixB and PAMFix, respectively, were obtained from Platelet Services (CRO. Bio City, Nottingham, UK). For the VASP phosphorylation measurements, the VASPFix was also obtained from Platelet Services. 

### 2.2. Methods

#### Blood Collection

This project received approval from the Research and Ethics Committee of Hospital Virgen de la Arrixaca (Murcia, Spain). Venous blood was obtained following informed consent from healthy volunteers who denied taking any medication in the preceding 14 days. Blood was taken by forearm venipuncture using a polypropylene syringe and a 19 G needle. Platelets were stabilized by performing a 30 min shake-free incubation at 37 °C before performing the experiment. For studies of platelet aggregation, P-selectin, and VASP phosphorylation, blood was taken into tri-sodium citrate dihydrate (3.13% *w*/*v*).

### 2.3. Platelet Aggregation

Aliquots (495 μL) of whole blood (that had been maintained at 37 °C) were dispensed into polystyrene LP3 tubes (64 × 11 mm) containing a stirrer bar and the different agents that were evaluated: saline (for the “starting” platelet count); TRAP-6 (10 μM); PGE3 (1 μM); and the EP3 and EP4 antagonists DG-041 (3 μM) and ONO-AE3-208 (1 μM), respectively. The tubes were placed in the stirring wells of a Multi-Sample Agitator (MSA, University of Nottingham, Nottingham, UK) operating at 1000 rpm and 37 °C. After a 4 min period, an aliquot of the blood was mixed with AGGFixA in the ratio of 1 part of the latter to 3 parts of blood. After 20 min, the sample (blood + AGGFixA) was diluted with AGGFixB at a ratio of 1 part of the AGGFixA treated sample with 9 parts of AGGFixB.

For analysis of platelet aggregation, an aliquot (5 μL) was added to a FACS tube containing 5 μL each of CD61-PerCP. All tubes were then incubated in the dark at 4 °C for 25 min. FACSflow (0.5 mL) was added to each sample immediately before reading on a BD FACSCanto^TM^ flow cytometer (BD Headquarters, Franklin Lakes, NJ, USA). Data were acquired with BD FACSDiva^TM^ acquisition software 6.0. A total of 50,000 red cells events were counted as internal control and CD61-positive platelet events were recorded. 

Platelet aggregation was calculated as the percentage loss of single platelets compared to the ‘starting’ platelet count (saline: without TRAP-6 or any other agent) of whole blood. When the effects of a particular antagonist were being determined, the antagonist was added to the blood prior to the other agents.

### 2.4. P-Selectin (CD62P)

Aliquots (20 μL) of TRAP-6 (final concentration of 10 μM) were added to microtubes, each containing 10 μL EDTA (final concentration 2 mM, to prevent aggregate formation) and 20 μL of PGE3 (final concentration of 1 μM). The tubes were then placed in a Multi Sample Agitator (operating in a non-stirring mode) at 37 °C for 4 min. Aliquots containing 450 μL of citrated whole blood that had been maintained at 37 °C were added, and the tubes were incubated for a further 4 min without stirring, after which 1 mL of PAMFix fixing solution was added to each tube. For analysis, an aliquot (5 μL) was added to a FACS tube containing 5 μL each of antibodies to CD61-PerCP and CD62P-FITC. All tubes were then incubated in the dark at 4 °C for 25 min. FACSflow (0.25 mL) was added to each sample immediately before reading on a BDFACSCantoTM flow cytometer. Data were acquired with BD FACSDiva^TM^ acquisition software 6.0. A total of 3000 CD61-positive platelet events were recorded. CD62P was quantitated as median fluorescence values (mf) for the whole population of platelets.

### 2.5. Measurement of VASP Phosphorylation

Aliquots (495 μL) of whole blood (that had been maintained at 37 °C) were dispensed into polystyrene LP3 tubes (64 × 11 mm) containing a stirrer bar and the different agents that were evaluated: saline (for the “starting” platelet count); TRAP-6 (10 μM); PGE3 (1 μM); and the EP3 and EP4 antagonists: DG-041 (3 μM) and ONO-AE3-208 (1 μM), respectively. The tubes were placed in the stirring wells of a Multi-Sample Agitator (MSA, University of Nottingham, Nottingham, UK) operating at 1000 rpm, 37 °C. After a 4 min period, an aliquot of the blood was mixed with VASPFix fixative solution a ratio of 5:1. After the addition of VASPFix, the samples were vortexed (to ensure cell lysis) and then incubated in the dark at room temperature for 2 h prior to analysis by flow cytometry.

Alternatively, they can be capped or sealed and immediately frozen for analysis at a later time point (recommended within 6 months). In the latter case, the samples should be removed from the freezer and incubated in the dark at room temperature for 2 h prior to flow cytometric analysis.

The VASP-P is quantitated on coated beads with VASP and VASP-P adsorbed on their surface and with anti-VASP-P antibody bound to the phosphorylated form of the protein (conjugated to a FITC fluorochrome). The beads themselves have APC fluorescence and can be easily identified.

Prior to the analysis of each tube, diluent buffer (300 μL) was added and vortexed thoroughly for 3–5 s just prior to flow cytometric analysis. The FITC Median Fluorescence (MF) of the acquired 300 beads was collected and recorded. Capture beads were identified by their associated APC fluorescence. Phosphorylated VASP was detected as FITC fluorescence expressed as median fluorescence (mf).

### 2.6. Data Presentation

Data are presented as mean ± SEM. Differences between mean values were determined using one-way ANOVA (Tukey’s multiple comparison test) using GraphPad Prism Software (GraphPad Version 10.3.1 (464), San Diego, CA, USA). The *p* values of 0.05 were considered significant. Actual numbers of experiments performed are provided in the Figure legends.

## 3. Results

We chose TRAP-6 as the primary agonist because this agonist, at the concentration used, was found to produce a maximal aggregation response in the majority of blood samples studied such that the potential for inhibition of PGE3 could be detected. 

### 3.1. TRAP-6-Induced Platelet Aggregation

**Figure 1:**
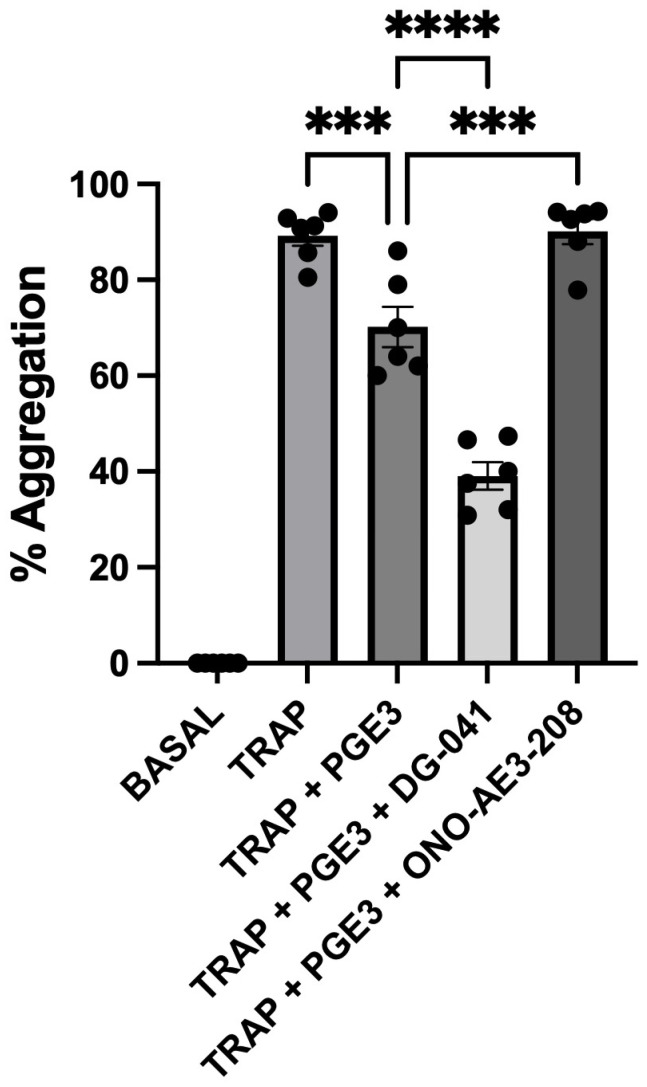
Effects of selective prostanoid receptor antagonists on the PGE3-mediated changes in TRAP-6-induced platelet aggregation. The effects of PGE3 in the absence and presence of the EP3 antagonist DG-041 (3 μM), and the EP4 antagonist ONO-AE3-208 (1 μM) on platelet aggregation induced by TRAP-6 (10 μM), over a 4 min period, in whole blood. Aggregation was determined by flow cytometry and measured by single platelet counting method. The results shown are the mean ± SEM of 6 experiments. *** *p* = 0.0004, **** *p* < 0.0001.

Experiments were conducted to determine the effect of PGE3 on TRAP-6-induced platelet aggregation and the ability of the EP3 antagonist, DG-041, and the EP4 antagonist, ONO-AE3-208, to modify this. TRAP-6 was used at a concentration of 10 μM, which was found to produce a high response compared with that produced by a range of different platelet agonists that we have studied. Here, the effect of PGE3 alone was to cause inhibition of aggregation. All antagonists were used at a concentration known to produce effective inhibition of the corresponding receptor. The effect of DG-041 (3 μM) was to enhance the inhibitory effect of the PGE3. In contrast to the effect of the EP3 antagonist DG-041, the effect of the EP4 antagonist ONO-AE3-208 (1 μM) was to potentiate platelet aggregation (Figure 1).

### 3.2. TRAP-6-Induced P-Selectin Expression

We chose measurements of TRAP-6-induced P-selectin to examine the effects of PGE3 on the secretory activity of platelets and the ability of the EP3 antagonist, DG-041, and the EP4 antagonist, ONO-AE3-208, to modulate the effects of PGE3 on this. TRAP-6 was used at a concentration of 10 μM, which was found to produce a high response compared with that produced by a range of different platelet agonists that we have studied. All antagonists were used at a concentration known to produce effective inhibition of the corresponding receptor. 

**Figure 2:**
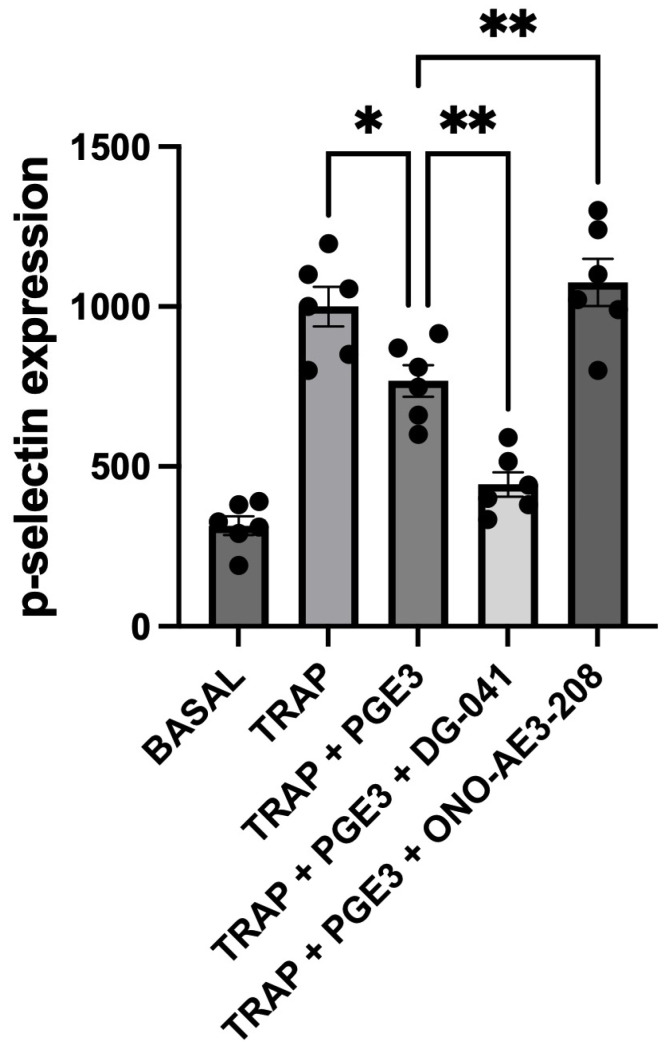
Effects of selective prostanoid receptor antagonists on PGE3-mediated changes in TRAP-6-induced P-selectin expression. The effects of PGE3 in the absence and presence of the EP3 antagonist DG-041 (3 μM), and the EP4 antagonist ONO-AE3-208 (1 μM), on platelet P-selectin expression induced by TRAP-6 (10 μM), over a 4 minute period, in whole blood. P-selectin was measured by flow cytometry and is presented as median fluorescence (mf). The results shown are the mean ± SEM of 6 experiments. * *p* < 0.05, ** *p* < 0.005.

TRAP-6 brought about a clear increase in P-selectin expression. In this context, the presence of PGE3 alone, at 1 μM, had little inhibitory effect on TRAP-6-induced P-selectin expression. However, in the presence of the EP3 antagonist DG-041, the inhibition brought about by PGE3 was clearly enhanced. In contrast, in the presence of the EP4 antagonist ONO-AE3-208, PGE3 promoted platelet P-selectin expression (Figure 2).

### 3.3. TRAP-6-Induced VASP Phosphorylation

Experiments were conducted to determine the ability of the EP3 antagonist, DG-041, and the EP4 antagonist, ONO-AE3-208, to modulate the effects of PGE3 on TRAP-6-induced platelet VASP phosphorylation. All antagonists were used at a concentration known to produce effective inhibition of the corresponding receptor.

TRAP-6 produced little effect on VASP phosphorylation. The effect of PGE3 alone was to bring about an increase in VASP-phosphorylation.

**Figure 3:**
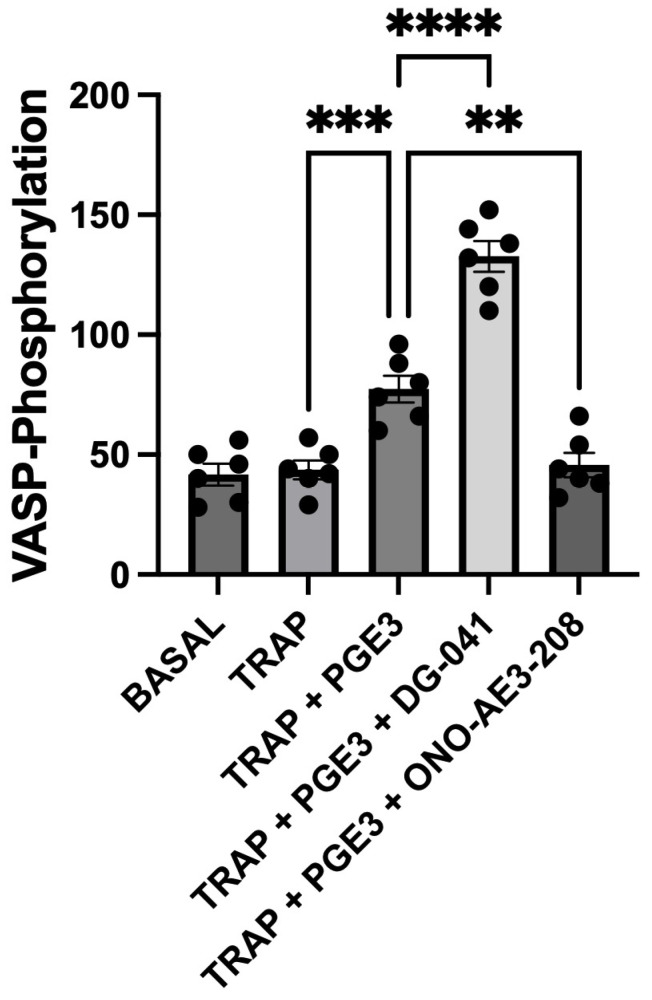
Effects of selective prostanoid receptor antagonists on the PGE3 mediated changes in TRAP-6-induced VASP-phosphorylation. The effects of PGE3 in the absence and presence of the EP3 antagonist DG-041 (3 μM), and the EP4 antagonist ONO-AE3-208 (1 μM) on VASP phosphorylation, over a 4 min period, in whole blood. VASP-phosphorylation was determined by flow cytometry using a cytometric bead array (VASPFix) and is presented as median fluorescence (mf). The results shown are the mean ± SEM of 6 experiments. ** *p* < 0.005, *** *p* = 0.0008, **** *p* < 0.0001.

The effect of the EP3 antagonist DG-041 was to potentiate PGE3-induced VASP-phosphorylation. Conversely, the effect of the EP4 antagonist ONO-AE3-208 was to markedly reduce VASP-phosphorylation brought about by PGE3 (Figure 3). 

## 4. Discussion

Previous studies have demonstrated that the overall effects of the prostaglandins of the E-series represent a balance between potentiation and inhibition of platelet function via interaction with Gs-coupled and Gi-coupled receptors. In this regard, there is evidence that PGE1 interacts with the IP (Gs-coupled receptor) and the EP3 (Gi-coupled receptor) receptors, although the overall effect is quite inhibitory, suggesting a stronger interaction between PGE1 and the IP receptor [24]. On the other hand, it has been extensively reported that PGE2 interacts with the EP4 (Gs-coupled receptor) and the EP3 (Gi-coupled receptor) receptors, resulting in a much more moderate effect on platelet function compared to PGE1, suggesting a tight balance between the two receptors [23]. A very similar scenario can be found with PGE3, where there is also an interaction between the EP4 and EP3 receptors, with moderate effects on platelet function, suggesting a tight balance between the two receptors.

That is the reason why the modulation of the EP receptors can determine the effects of prostaglandin on platelet function. For instance, in the presence of DG-041, an EP3 antagonist, the overall effect of PGE2 is an EP4-dependent inhibition of platelet function, even at a low concentration [23,24]. This inhibition can be further potentiated in the presence of P2Y12 antagonists, suggesting the important role that the activation of adenylate cyclase plays on the inhibition of platelet function [26]. 

Since variants of the gene encoding for the EP3 receptor for the PGE2 that approximately double the risk of suffering from peripheral artery disease were identified, the company deCODE genetics developed a novel, first in class antagonist of the EP3 receptor as an anti-platelet drug [7]. Following deCODE’s gene discovery, a manuscript published by Fabre et al. [10] demonstrated in mice that PGE2 is produced in atherosclerotic plaques, promoting, through the EP3 receptor, the formation of clots immediately at the sites of plaque but not over normal blood vessels, opening new pathways in a manner different to TXA2 synthesis inhibitors or P2Y12 receptor antagonists, for the treatment of arterial thrombosis. In this scenario, DG-041 was evaluated in phase Ib or II studies, although further developments seem to have been discontinued [34].

However, beyond the pharmacological modulation and blockage/activation of the EP3 or EP4 receptors, the balance of the natural expression between EP3 and EP4 receptors may play a pivotal role in the cardiovascular system, since it has been reported that the overexpression of EP3 receptor reduces cardiac function under basal conditions and that this is exacerbated after myocardial infarction [27]. In addition, other investigators have published that the atherothrombotic complications associated with hyperglycemia/diabetes may be mediated via a potentiation of the platelet activation and arterial thrombus formation via PGE2/EP3 signaling, since the blockade of the EP3 receptor activation reversed the hyperactivity of platelets and delayed thrombus formation in hyperglycemic mice [35]. In contrast, the overexpression of EP4 improves cardiac function after a myocardial infarction [28]. In fact, previous studies have suggested that the activation of the EP4 receptor in platelets could be a good novel antiplatelet strategy [36]. Also, it has been reported that endothelial EP4 receptor promotes nitric oxide in endothelial cells and vasodilation, suggesting that EP4 activation may represent a new strategy to treat hypertension [37]. Other authors have published that mPGES-1, a therapeutic target downstream of COX enzymes, protects from cardiac ischemia/reperfusion injury, limiting leukocyte–endothelial interactions and preserving microvascular perfusion partly via the endothelial EP4 receptor [38]. These naturally occurring changes on the expression of EP3/EP4 receptors may influence the ability and efficacy of prostaglandins to regulate the cardiovascular system and modulate platelet function.

As mentioned earlier, cardiovascular diseases rank as the primary global causes of mortality, significantly impacting overall well-being. In this regard, ischemic heart disease and ischemic stroke are leading the global rank of the cardiovascular deaths by cause [1]. In both situations, the involvement of platelet activation plays a pivotal role in the pathophysiology of the process [2]. In this context, several clinical trials have consistently shown that icosapent ethyl, a highly purified ethyl ester of EPA, reproducibly reduced cardiovascular events and the progression of atherosclerosis [9]. In that context, the REDUCE-IT study has revealed a cardiovascular risk reduction with icosapent ethyl [15]; the EVAPORATE study has confirmed that icosapent ethyl is able to produce a significant regression of atherosclerotic plaque volume [16]; and JELIS has proved a reduction in cardiovascular events in hypercholesterolemic patients with EPA ethyl ester added to statin therapy [17]. All these beneficial cardiovascular effects are conferred through a broad pleiotropic mechanism that can protect from endothelial dysfunction, plaque vulnerability, lipoprotein oxidation, inflammation, or ischemic events [2].

Other studies have revealed that omega-3 fatty acids may enhance the antiplatelet effect of prostaglandins, confer cardioprotection against arrhythmias [18], and lower rates of fatal myocardial infarction and sudden cardiac death [14]. In addition, resolving E1, an omega-3 fatty acid-derived compound, is capable of regulating collagen-induced platelet aggregation [22]. However, it has also been reported that several clinical trials have showed no significant benefit of omega-3 fatty acid supplementation on cardiovascular disease events, as is the case in the manuscript published by Aung T et al. in 2018, which reported the meta-analysis of 10 trials prior to REDUCE-IT involving 77,917 individuals [39]. Similar results were obtained in the STRENGTH trial, showing no reduction in cardiovascular events with an Eicosapentanoic Acid (EPA)/ Docosahexanoic Acid (DHA) mixed formulation in a similar patient population as REDUCE-IT [40].

These discrepancies in the outcomes of all these clinical trials may depend on different circumstances, which could be related to the capacity of the different omega-3 fatty acid compounds to generate prostaglandins, like PGE3 or other similar compounds such as resolvins or maresins. Simultaneously, the diverse pathological conditions of individuals involved in these trials can result in a wide range of expression levels for various molecules and/or receptors, like EP3 or EP4 receptors. These factors may impact prostaglandin’s ability to regulate the cardiovascular system and platelet function, consequently influencing the outcomes of the clinical trials. 

In this study, we investigated the ability of PGE3 at 1 μM, that is, a high concentration with the tendency to inhibit platelet function, to inhibit TRAP-6-induced platelet reactivity and at the same time the capacity of different EP receptor antagonists, namely EP3 receptor antagonist DG-041 and the EP4 receptor antagonist ONO-AE3-208, to modify the effects of PGE3 on platelet function. We have used TRAP-6 as the main agonist since thrombin stimulates the formation of procoagulant (phosphatidylserine-exposing) and coated (fibrin-binding) platelets, which leads to the amplification of thrombin generation that is a key element for thrombus formation [30]; therefore, exploring the ability of PGE3 to modulate the effects of thrombin could be very relevant in vivo.

PGE3 (1 μM) brought about weak inhibition of TRAP-6-induced platelet aggregation and P-selectin expression. This inhibition was enhanced in the presence of the EP3 receptor antagonist DG-041, suggesting that, when the EP4 receptor becomes the main available receptor, the balance between EP3 and EP4 shifts to EP4, resulting in a much stronger inhibition of platelet reactivity by PGE3. These results may indicate that, under natural conditions in which EP4 receptors are overexpressed [28,37,38], or the EP3 receptor is underexpressed or blockaded, the ability of PGE3 to inhibit platelet function may be enhanced. In contrast, in the presence of the EP4 antagonist ONO-AE3-208, the inhibitory effect of PGE3 was abolished.

**Figure 4:**
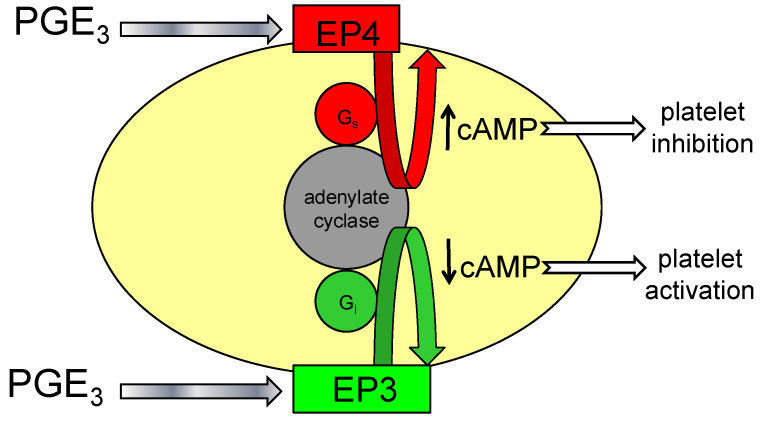
Diagrammatic view of the receptors and their consequent effects on adenylate cyclase, which mediates the effects of PG3 on platelet function (modified from Iyú D et al. [24]).

Therefore, the availability of the EP4 is essential for PGE3 to inhibit platelet reactivity (Figure 4). Our results indicate that PGE3 has the potential to inhibit platelet aggregation but also P-selectin expression. In this regard, the ability to inhibit P-selectin expression could be very important in vivo, since this molecule is the marker of the release of alpha granules that contain very important mediators for promoting coagulation and inflammation [41]. Also, P-selectin is the molecular bridge that facilitates the interaction between platelets and leukocytes, generating platelet–leukocyte aggregates, resulting in inflammation [42] and the expression of tissue factor on monocytes [43]. Therefore, many of the beneficial effects for the cardiovascular system that have been described for omega-3 fatty acids may be mediated by PGE3, at least in certain circumstances. 

The effects of PGE3 on platelet function, like the other prostaglandins of the E-series, are mediated by Gs- and Gi-coupled receptors that activate and inhibit, respectively, adenylate cyclase, an enzyme that regulates the intracellular level of cAMP. Since the levels of phosphorylated vasodilator-stimulated phosphoprotein (VASP) can be used as a surrogate marker of changes in cAMP levels in platelets [25,26,31,32], we have used this approach in this study to evaluate the effects of PGE3 in cAMP levels. 

Our results indicate that PGE3 brought about an increase in VASP phosphorylation. This increase was clearly enhanced in the presence of the EP3 receptor antagonist DG-041, suggesting that this increase in VASP phosphorylation may be related to the potentiation of the ability of PGE3 to inhibit TRAP-6-induced aggregation and P-Selectin expression. In contrast, in the presence of the EP4 receptor antagonist ONO-AE3-208, the increase in VASP phosphorylation was abolished, suggesting that when the EP4 receptor is blocked, PGE3 counteracts more efficiently in cAMP through its interaction with EP3 receptors, which could be related to the incapacity of PGE3 to inhibit TRAP-6-induced aggregation and P-selectin expression, indicating that the EP4 receptor is the leading pathway that mediates the increase in cAMP and therefore the ability of PGE3 to inhibit platelet reactivity. 

This capacity of PGE3 to activate adenylate cyclase through the EP4 receptor (Gs-coupled receptor), once the EP3 receptor is blocked or underexpressed, may be very important in vivo, since it can potentiate the actions of other naturally occurring modulators of platelet aggregation that act via cAMP, like PGE1, PGE2, PGD2, prostacyclin (PGI2), or adenosine [26]. Interestingly, this scenario might be very similar to the situation where the P2Y12 receptor, a Gi-coupled receptor like the EP3 receptor, is blocked by P2Y12 receptor antagonists [44], since the blockade of P2Y12 may promote and enhance the inhibition of platelet function brought about by compounds that raise cAMP, like prostaglandins [26] or adenosine [45]. Additionally, that inhibition can be further enhanced when P2Y12 and EP3 receptors are blocked at the same time, suggesting the important role that adenylate cyclase may play on the regulation of platelet function [26].

Taking all these aspects into account, we may argue that the consequences and outcomes of the supplementation of, consumption of, or clinical trials that use omega-3 fatty acids might depend on numerous factors related to the type of omega-3 used, the level of expression or activity of EP3/EP4/P2Y12 receptors, the ability to generate PGE3 or similar compounds like resolvins, and many others, and this warrants further investigation.

## 5. Conclusions

In conclusion, our results suggest that the overall effects of PGE3 on human platelet function are the consequence of a balance between the promotor effects of the prostanoid acting at the EP3 receptor and its inhibitory effects at the EP4 receptor. The occupation of EP4 receptors leads to the inhibition of platelet function, brought about by thrombin, via increasing the level of cAMP, and indicates that the inhibitory effects of PGE3 are mediated by this receptor. In contrast, the interaction with the EP3 receptor may potentiate platelet function by lowering cAMP. In general, the ability of PGE3 to inhibit thrombin-induced platelet reactivity may be one the mechanisms by which the cardiovascular benefits of dietary omega-3 fatty acids are conferred.

## Data Availability

The original contributions presented in the study are included in the article, further inquiries can be directed to the corresponding author.
